# Obesity induced transcriptional changes in skeletal muscle across different species

**DOI:** 10.1371/journal.pone.0327988

**Published:** 2025-07-14

**Authors:** Yujie Wang, Jiaman Zhang, Xintong Yang, Fuwen Wang, Long Jin, Jing Li, Xuewei Li, Mingzhou Li

**Affiliations:** 1 Livestock and Poultry Multi-omics Key Laboratory of Ministry of Agriculture and Rural Affairs, College of Animal Science and Technology, Sichuan Agricultural University, Chengdu, China; 2 Yunnan Province Key Laboratory for Porcine Gene Editing and Xenotransplantation, Yunnan Agricultural University, Kunming, China; 3 Key Laboratory of Animal Genetics, Breeding and Reproduction of Shaanxi Province, College of Animal Science and Technology, Northwest A&F University, Yangling, China; South China Agricultural University, CHINA

## Abstract

Pigs on high-fat diets maintaining metabolic homeostasis and are resistant to hepatic steatosis, differing from humans and mice. Obesity-induced metabolic dysregulation and inflammation in skeletal muscle are well-studied in humans and mice, but less is known about pig skeletal muscle responses. This study constructs the skeletal muscle transcriptome of obese pigs and integrates it with publicly available transcriptional profiles from obese humans and mice, and ATAC-seq data from lean individuals across species. We systematically characterized transcriptional changes in skeletal muscle under stress of obesity, focusing on the evolution of gene families, orthologous genes, and epigenetic regulation. Our results show that obesity activates lipid catabolism genes and inhibits immune response genes in pig skeletal muscle, contrasting with humans and mice. We identify expanding gene families in pigs, such as olfactory receptors, α-amylase, and ABC transporters, which are upregulated in obesity. While oxidative metabolism-related gene families are contracted in the human and mouse genomes and are downregulated with obesity. By comparing orthologous genes, we identify a set of divergently changing genes induced by obesity across species, which primarily participate in lipid metabolism, inflammation, and immune cell activation. High-divergence genes show conserved coding and promoter sequences, and exhibit greater chromatin accessibility in promoter regions, compare with low-divergence genes. These findings suggest that gene dosage and transcriptional plasticity contribute to species-specific expression divergent responses to obesity. Identifying rapidly evolving gene families, divergently expressed genes, and potential transcription factor binding sites may reveal new insights into obesity-related metabolic disorders and therapeutic targets.

## Introduction

Obesity is a public health issue worldwide [1], and is a major risk factor for various metabolic diseases, such as cardiovascular diseases, diabetes, and metabolic syndrome [2,3]. Environmental factors, particularly the consumption of a fat-rich Western diet or high-fat diet, play a significant role in the progression of obesity [4]. Current research on obesity mainly focuses on liver steatosis [5,6] and the pro-inflammatory effects of hypertrophic adipocytes [7,8]. However, skeletal muscle, as the largest metabolic organ in the body, plays a key role in maintaining metabolic homeostasis [9]. Therefore, the impact of obesity on skeletal muscle is also deserving of attention. Previous research has demonstrated that the lipolytic capacity of skeletal muscle is reduced in obese individuals, which leads to ectopic fat accumulation, and subsequently promotes chronic low-grade inflammation and metabolic dysregulation throughout the whole body [10–12].

Pigs are the preferred large animal model for metabolic studies, as their metabolic characteristics, cardiovascular system, and omnivorous habits are similar to those of humans [13,14]. However, pigs seem to have undergone adaptive evolution to high-energy diets during long-term domestication [14–17]. For example, our previous studies reported that a 22-week high-fat diet induced typical obesity characteristics in Bama miniature pigs, including excess weight, adipocyte hypertrophy, and elevated blood lipids [18,19]. However, obesity induced only minimal inflammation and an almost normal liver morphology [18]. Inducing the progression from obesity to a diabetic phenotype in pigs can require specific interventions, such as streptozotocin treatment to destroy pancreatic cells [20], or genetic modifications targeting key genes, including pancreatic GIPR [21] that regulates insulin release, and hepatic HNF1α [22] that mediates β-cell development. This highlights that pigs are able to maintain metabolic homeostasis and resist further metabolic dysregulation despite being obese (2.03-fold weight gain in our obesity model [18]). In contrast, most humans with type 2 diabetes (85% [23]) have a BMI of 25 kg/m² or higher, while mice exhibit systemic metabolic dysregulation with only a 1.6-fold increase in body weight [24]. These reports suggest that pigs might be adapting to a “diabetes-prone” environment (*i.e.,* adequate food and low-exercise captive lifestyle) during domestication.

In this study, we profile the skeletal muscle transcriptome employing our previously established high-fat diet-induced obese pig model. We then integrate these data with publicly available skeletal muscle transcriptome datasets from obese patients and diet-induced obese mouse models, as well as muscular ATAC-seq (assay for transposase-accessible chromatin sequencing) data from lean pigs, mice, and humans. This allows us to conduct a systematic study of the divergent transcriptional regulation of skeletal muscle across species in response to obesity stress, from the perspective of the evolution of gene families, orthologous genes, and epigenetic regulation.

## Materials and methods

### Ethical statement

All pig experiments in this study were conducted in accordance with the ethical guidelines and regulations for the care and use of laboratory animals. The experimental protocol was reviewed and approved by the Institutional Animal Care and Use Committee in the College of Animal Science and Technology, Sichuan Agricultural University, Sichuan, China, under permit No. DKY-2019102015. All pigs were effectively stunned by electrical means prior to sampling, followed by humane slaughter through exsanguination to minimize pain and distress. All procedures were carried out in accordance with established animal welfare guidelines, ensuring ethical treatment and respect for animal life throughout the experimental process.

### Pig muscle collection and RNA-seq library construction

This study used the high-fat diet-induced obese pig model previously characterized by our group [18,19]. Briefly, all pigs were housed under standard conditions with controlled temperature, humidity, and a 12-hour light or dark cycle, and they were provided with *ad libitum* access to food and water. Adult pigs (two years old) were fed a high-fat diet for 22 weeks, leading to significant increases in body weight, adipocyte size, and blood lipids compared with pigs fed with standard diet. Skeletal muscle samples from the *longissimus dorsi* muscle (*LDM*) and *psoas major* muscle (*PMM)* were immediately flash-frozen in liquid nitrogen upon collection and stored at −80°C for subsequent RNA extraction.

Total RNA was extracted from pig muscle samples through the MiniBEST Universal RNA Extraction Kit, following the manufacturer’s instructions. The RNA yield and integrity (RIN) were assessed using a Qubit and Agilent 2100 Bioanalyzer. Only RNA samples with a total yield of more than 3 µg and a RIN score above 8 were considered suitable for transcriptome sequencing. The poly(A) enrichment strategy was employed to construct RNA libraries, which were subsequently sequenced on a 150-bp paired-end model on the DNBSEQ-T7 platform.

### Quantification of gene expression abundance and DEGs identification

Trimmomatic [25] (v 0.4) was used to remove reads with more than 20% of low-quality bases (Phred Quality Score < 20). High-quality reads were aligned to the transcripts (Ensembl 106) of humans, mice, or pigs using Kallisto [26] (v 0.44.0) to obtain gene count numbers and Transcripts Per Million (TPM) values. For the pig data, limma [27] was used to correct for potential batch effects (S1 Table). Differentially expressed genes (DEGs) were identified using edgeR [28] (v 3.20). Genes with a *p*-value < 0.05, | log_2_(fold change) | > 1, and TPM > 0.25 in at least half of the biological replicates were considered significantly differentially expressed.

Gene Set Enrichment Analysis (GSEA) was performed to evaluate the gene expression change trends in metabolic disorder, inflammatory response, and immune response-related pathways between obese and lean individuals across pigs, mice, and humans. The analysis was conducted using GSEA [29] software (v 4.3.3) with gene sets obtained from the Gene Ontology database, specifically GO:0019433, GO:0098803, GO:0006954, and GO:0002253. Genes were ranked based on their log_2_(fold change) values, and the enrichment score (ES) was calculated using a weighted Kolmogorov-Smirnov-like statistic. The core enrichment genes are those in the gene set that appear in the ranked list before the enrichment score reaches its peak. Gene sets with a *p*-value < 0.05 were considered significantly enriched.

### Identification of rapidly evolving gene families

To identify rapidly evolving gene families, protein sequences from 11 species (rat, mouse, hamster, macaque, chimpanzee, human, horse, pig, cow, sheep, and elephant) were collected from Ensembl (version 106). OrthoFinder [30] (v2.5.4) was used to identify orthologous gene families across these species through an all-against-all BLASTP alignment. Significant expansion (or contraction) of gene families in a given species was identified based on *p*-values < 0.05 calculated by CAFÉ [31] (v3.1) and copy numbers greater (or lower) than the median of the other species.

To determine the functions of the expanded gene families upregulated in obese pigs, we used InterProScan [32] to search for functional domains in the protein sequences of each gene copy, based on the Pfam [33] database. The most frequent domain was considered the representative functional domain for each gene family.

### Identification of obesity-induced divergent expression change genes across species

We filtered 1:1:1 orthologous genes among pigs, mice, and humans and retained those that exhibited differential expression between obese and lean individuals in at least one species. After filtering, a total of 773 single-copy genes were retained for further analysis. The standard deviation of log_2_ fold change (log_2_FC, the expression change between obese and lean individuals in a given species) across the three species was then calculated. Since small fluctuations in lowly expressed genes can produce disproportionately large fold changes (S1A Fig), they may unduly inflate the standard deviation of interspecies log_2_FC (S1B Fig). For example, although the expression level of the GDA gene was below 0.5 in both lean and obese pigs, it nevertheless resulted in a fold change greater than 1 (S1C Fig). The standard deviation of its log_2_FC across the three species was as high as 0.77 (S1C Fig). To reduce the influence of such low expression genes on fold change variability, we applied an empirical Bayes shrinkage approach to the counts normalized by the median-of-ratios method (S1D Fig). Additionally, for genes with low expression levels (TPM ≤ 0.25 in at least half of the biological replicates), log_2_(fold change) values were set to zero to further reduce noise from low-abundance transcripts. This strategy effectively attenuated the impact of noise from lowly expressed genes on the overall standard deviation (S1 Fig). Furthermore, we evaluated the correlation of log_2_FC standard deviations calculated using counts from different normalization methods. The results confirmed the robustness of our normalization strategy (Pearson’s *r* > 0.99) (S1E Fig). Subsequently, based on the density curve of the standard deviation of log_2_(fold change) for these 773 genes, we classified the genes into three categories: high (top 25%), medium (middle 50%), and low (bottom 25%) divergence in expression change.

### Analysis of genomic features for genes with divergent changes

To quantify the selective pressure on genes with divergent changes, we downloaded the protein sequences (Ensembl, version 106) of single-copy homologous genes from pigs, mice, humans, and an outgroup (elephant). To minimize alignment-related errors, we first aligned the protein sequences using MUSCLE [34] (v 3.8.31), The resulting alignments were then converted into codon alignments using PAL2NAL [35]. Finally, the branch-site model implemented in PAML (v 4.7) [36] was used to estimate the ratio of nonsynonymous to synonymous substitutions (dN/dS or ω).

To further investigate the evolutionary mechanisms underlying transcriptional divergence, we analyzed the features of the promoter regulatory regions of divergent genes. the human PhastCons7 score was obtained from UCSC Genome Browser, and used to assess the conservation of the promoter sequences by evaluating the nucleotide substitution rate across the human divergent gene promoter regions (+200 bp of the transcription start site (TSS)). The PhastCons scores of human divergent genes were aligned based on their TSS positions, and the median PhastCons score for each divergent genes were calculated for visualization. The TATA-box transcription factor binding motif was downloaded from the JASPAR [37] database (motif ID: MA0108.1) and used with FIMO [38] to search matching motifs in the promoter regions (+200 bp and −50 bp of the TSS) of divergent genes across three species. CpG island (CGI) annotation data was obtained from the UCSC Genome Browser (hg38, mm39, and sus11), and CGI genes were defined as those with promoter regions (+300 bp and −100 bp of the TSS) overlapping CGI.

### Processing of ATAC-seq data and syntenic OCRs Identification

After filtering out low-quality reads, high-quality reads were aligned to the reference genomes of pig (Sscrofa11.1), mouse (GRCm39), or human (GRCh38) using Bowtie2 [39] software (v 2.5.4). Mitochondrial alignments, low-quality alignments (Q < 20), and PCR duplicates were removed from the mapped reads using SAMtools [40] (v 1.21). Nucleosome position shifts and transposase cleavage biases were corrected using the ATACshift [41] tool. ATAC peaks were identified using MACS2 [42] (v 2.2.9.1) with a *p*-value < 0.05, and only those peaks overlapping the promoter regions (+2200 bp and −500 bp of TSS) of the divergent genes were retained. To minimize batch effects, such as sequencing depth, platform, and other systematic biases across datasets, we extended 250 bp upstream and downstream of each peak and normalized the open chromatin regions (OCRs) between species to a uniform length of 501 bp.

The Liftover [43] tool was used to align the OCRs of a given species to the homologous regions in the other two species. The OCRs were classified into three categories based on the homologous regions: 1) species-specific OCRs, which could not be aligned to the homologous promoter regions in the reference species; 2) usage-specific OCRs, which aligned to the homologous promoter regions but lacked OCRs in the reference species; and 3) usage-conserved OCRs, which aligned to the homologous promoter regions and exhibited OCRs in both species.

### Functional enrichment analysis

We performed functional enrichment analysis using Metascape [44] (http://metascape.org). The functional pig genes were mapped to their human symbols using the BioMart tool. Human symbols were used as input for the enrichment analysis. Human (*Homo sapiens*) was chosen as the target species, with all genes considered as the background set for the enrichment analysis. The results presented only the top five statistically significant GO terms.

### Transcription Factor Binding Site Enrichment Analysis

Enrichment analysis of transcription factor binding motifs was performed using the MEME Suite [45] (v 5.5.7). Briefly, genomic regions of interest were extracted in FASTA format. Background sequences were generated by shuffled sequences to preserve nucleotide composition while disrupting motif structure. The Analysis of Motif Enrichment (AME) tool [46] was employed to identify overrepresented motifs based on JASPAR 2022 vertebrate non-redundant motif database [47]. Statistical significance was assessed by Fisher’s exact test with *q*-value < 0.05.

## Results

### Obesity induces transcriptional reprogramming in pig skeletal muscle

To investigate the effect of obesity on gene expression in pig skeletal muscle, we selected the *longissimus dorsi* muscle (*LDM*, predominantly fast-twitch fibers [48]) and *psoas major* muscle (*PMM*, predominantly slow-twitch myofibers [48]) of lean (*n* = 9) and high-fat diet-induced obese pigs (*n* = 10) for transcriptome sequencing. A total of 38 poly (A)-enriched transcriptome libraries were generated, yielding ~899.9 million high-quality reads, 93.44% of which were uniquely mapped to the pig reference genome (S1 Table, Alignment statistics for sequencing datasets used in this study).

**Fig 1:**
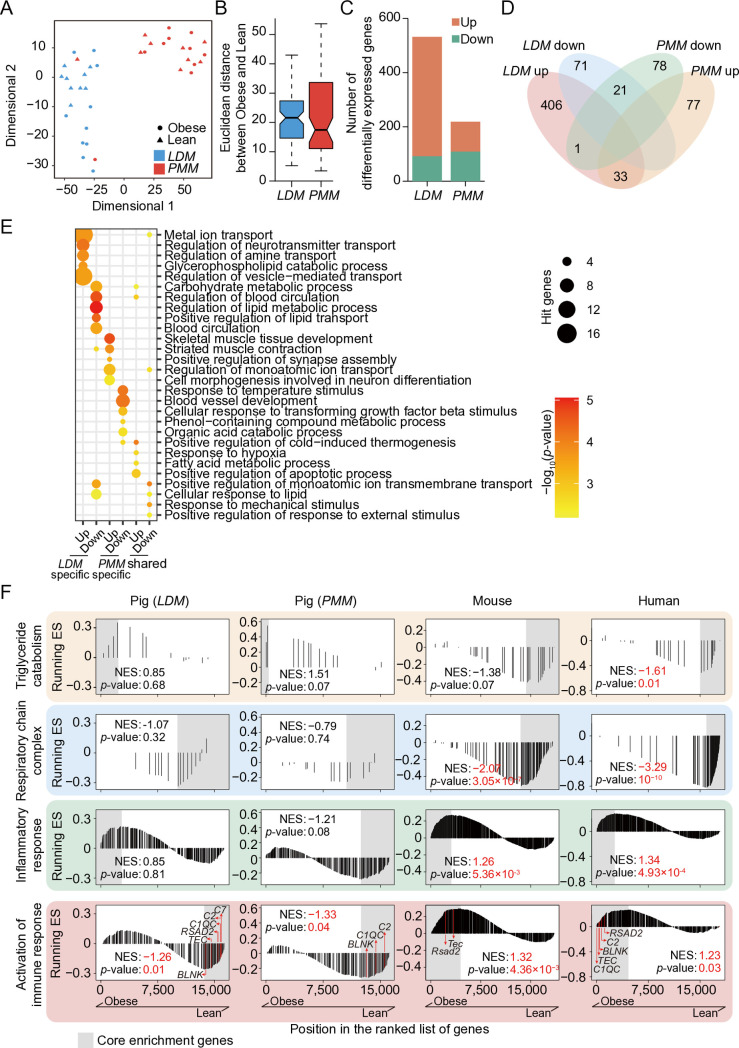
The transcriptomic profiles of skeletal muscle in lean and obese pigs. **A**: t-SNE dimensionality reduction of transcriptomic gene expression of skeletal muscle of lean and obese pigs. **B:** Boxplot of Euclidean distances between the *LDM* and *PMM* of lean and obese pigs, after excluding two obvious outliers in the *PMM* group. **C:** The number of differentially expressed genes (DEGs) between the obese and lean pigs of *LDM* and *PMM*. **D:** Venn diagram illustrating overlapping obesity-induced DEGs between the *LDM* and *PMM* muscles. **E:** The top five most significantly enriched Gene Ontology Biological Process (GO–BP) terms for *LDM*- and *PMM*-specific DEGs, and shared DEGs. **F:** GSEA analysis of genes related to energy metabolism, inflammation, and immune response in pig skeletal muscle (*LDM* and *PMM*), as well as in mouse and human skeletal muscle. Core enrichment genes, defined as those appearing before the peak of the running enrichment score in the GSEA plot, are highlighted with a grey background. t-SNE, t-distributed stochastic neighbor embedding; *LDM*, *longissimus dorsi* muscle; *PMM*, *psoas major* muscle; DEGs, differentially expressed genes; ES, enrichment score; NES, normalized enrichment score.

The t-distributed stochastic neighbor embedding (t-SNE) analysis clearly separated the *LDM* and *PMM*, reflecting the inherent differences between skeletal muscles with distinct metabolic properties (Fig. 1A). More importantly, we observed a greater impact of obesity on the transcription profile of *LDM* compared to *PMM*, the Euclidean distance between obese and lean subjects for *LDM* and *PMM* were 21.58 and 17.45, respectively (Fig 1A–B), and the number of differentially expressed genes (DEGs) between the obese and lean subjects further supports this trend (*LDM *vs.* PMM*: 532 *vs.* 210) (Fig. 1C). The majority of obesity-induced DEGs (~92%) were muscle group-specific (Fig. 1D). *LDM*-specific DEGs were mainly involved in pathway related to metabolism and transport of substrates (*e.g.,* ‘carbohydrate metabolism’, ‘lipid metabolism’, and ‘lipid transport’) (Fig. 1E). Moreover, *PMM*-specific DEGs were primarily enriched in muscle homeostasis-related pathways (*e.g.,* ‘muscle development’, ‘muscle contraction’, and ‘angiogenesis’) (Fig. 1E). Conversely, about 8% of obesity-induced DEGs were shared between *LDM* and *PMM*. These genes were mainly enriched in pathways related to stress response and metabolism (*e.g.,* ‘apoptosis’, ‘hypoxia response’, ‘lipid response’ and ‘fatty acid metabolism’) (Figs 1D–E). These results suggest that obesity alters the expression of metabolism-related genes, and activates the expression of stress response genes in pig skeletal muscle. Notably, the impact of obesity is more pronounced on fast-twitch muscles, such as *LDM*.

Notably, we did not observe the upregulation of inflammation- and immune-related genes in pig muscle, as reported in obese patients and diet-induced obese mice [49,50] (S2 Fig, publicly available data; S1 Table). To assess the expression changes of the ‘obesity-associated’ genes in skeletal muscles with different muscle fiber compositions in pigs, as well as in fast-twitch muscles across species, we performed gene set enrichment analysis (GSEA) to assess the transcriptional dynamics of genes involved in lipid degradation, mitochondrial respiration, inflammation, and immune response in these datasets (Fig. 1F; S2 Table, core gene annotation of ‘obesity- associated’ gene sets).

In both the *LDM* and *PMM* of pigs, obesity led to the upregulation of genes related to triglyceride degradation and the downregulation of genes associated with the mitochondrial respiratory chain (Fig. 1F). Although these changes did not reach statistical significance, we observed that, compared to *PMM*, the *LDM* exhibited a lower enrichment score for triglyceride degradation genes (*LDM* *vs. PMM*: 0.85 vs. 1.51) and a higher enrichment score for mitochondrial respiration genes (*LDM* *vs.* *PMM*: −1.07 vs. −0.79) (Fig. 1F). Fast-twitch muscles, such as the *LDM*, naturally have fewer mitochondria and capillaries than slow-twitch muscles [10], which could explain the lower lipolytic capacity and more serious mitochondrial dysfunction observed in *LDM* compared to *PMM* when exposed to high free fatty acid milieu associated with obesity. Additionally, we observed that obesity induced a mild upregulation of inflammatory response genes in the *LDM*, and slightly suppressed the expression of the inflammatory response genes in the *PMM* (Fig. 1F). Importantly, genes associated with the immune response were downregulated in both the *LDM* and *PMM* (Fig. 1F). Conversely, in mouse and human skeletal muscles, obesity led to a reduction in the expression of lipolysis- and mitochondrial function-related genes, as well as activation of genes associated with inflammation and immune response (Fig. 1F). For example, core genes such as *C1QC*, *C2*, and *C7* (involved in the complement system), and *BLNK*, *RSAD2*, and *TEC* (involved in cytokine signaling pathways), exhibited opposing expression changes between mice, humans, and pigs (Fig. 1F; S2 Table).

Taken together, obesity showed a more pronounced effect on fast-twitch muscles in pigs, leading to mild mitochondrial dysfunction and inflammation, while slightly upregulating lipolytic capacity and suppressing immune response. This contrasts with the metabolic dysregulation and activation of inflammation and immune responses observed in the fast-twitch muscle groups of obese humans and diet-induced obese mice.

### The role of rapidly evolving gene families in skeletal muscle with obesity across different species

**Fig 2:**
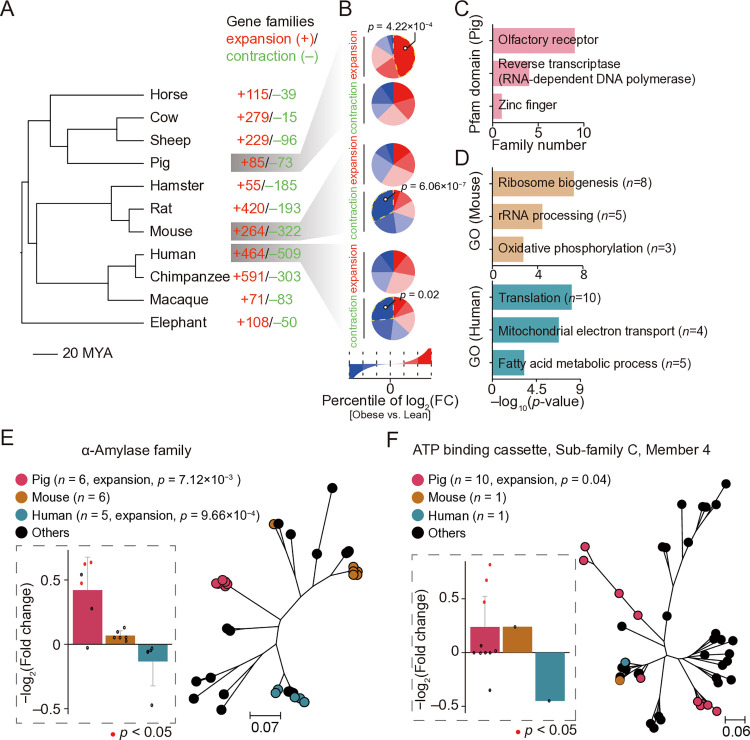
Rapid evolution of gene families contributes to shaping interspecies skeletal muscle obesity phenotypes. **A:** Phylogenetic relationships of eleven placental mammals, with gene families exhibiting significant expansion and contraction marked in red and green, respectively, on the nodes. **B:** The percentage of gene expression changes between obese and lean subjects with significant expansions and contractions of gene families in pigs, mice, and humans. The upregulated and downregulated genes were divided into three percentiles. **C:** Pfam annotation of the protein sequences for the obesity-induced upregulated expanded gene families in the pig genome. **D:** Functional enrichment of the obesity-induced downregulated contracted gene families in the mouse and human genomes. **E**–**F:** Obese-induced expression fold changes and phylogenetic tree of the α-amylase (**E**) and ABCC4 (**F**) gene families. Genes with significant expression changes (*p*-valu*e* < 0.05, calculated by edgeR) are highlighted with red points. The *p*-values in **B** and **D** pane were calculated by Fisher’s exact test and hypergeometric test, respectively.

Rapidly evolving gene families are key drivers in the acquisition of new functions or physiological traits [51,52]. In the context of the specific gene expression changes in pig skeletal muscle under obesity, rapidly evolving gene families may play a crucial role in the metabolic adaptation of pig skeletal muscle. We identified the expansion and contraction of gene families across pigs, mice, humans, and eight other placental mammals (Fig 2A), and examined the expression changes of gene copies in fast-twitch muscle groups between obese and lean subjects (Fig. 2B). We found that most gene copies (~45%) of the families expanded in pigs were upregulated in obese subjects (fold enrichment = 1.20, *p*-value = 4.22 × 10^−4^, Fisher’s exact test) (Fig. 2B). In contrast, in the gene families contracted in mice and humans, most gene copies (33% and 28% in mice and humans, respectively) were downregulated in the obese subjects (fold enrichment = 2.16 and 1.36, *p*-value = 6.06 × 10^−7^ and 0.02 for mouse and human, respectively, Fisher’s exact test) (Fig. 2B). These findings suggest that gene family expansions and contractions play a role in species-specific responses to obesity-related metabolic disease. However, only a few of the upregulated expanded gene families in the pig genome were annotated, including the α-amylase family and the ATP-binding cassette (ABC) transporter family. To gain a more comprehensive understanding of the functions of these expanded gene families with upregulated transcription, we aligned their protein sequences to the Pfam database [33] and identified the most prevalent functional domains. Notably, nine of these families were predicted to contain olfactory receptor domains (Fig. 2C). Several olfactory receptor families have been reported to regulate lipid metabolism [53,54], suggesting that pigs may modulate metabolic homeostasis in myocytes through olfactory receptor-mediated nutrient sensing. In contrast, the downregulated contracted gene families in mice and humans were primarily involved in protein translation and oxidative metabolism related biological processes (*e.g.,* ‘translation’, ‘oxidative phosphorylation’ and ‘fatty acid metabolism’).

For instance, the α-amylase gene family exhibits significant expansion in the genomes of both pig (six copies) and human (five copies) (**Fig. 2E**). As a classical digestive enzyme, amylase primarily catalyzes the breakdown of polysaccharides into simpler sugars. Obese patients exhibit reduced circulating amylase levels [55], this has been associated with an increased risk of metabolic syndrome [56]. A recent study of human amylase gene copy number further revealed that individuals with lower copy numbers of amylase genes were predisposed to obesity [57]. Intriguingly, amylases are also expressed in non-digestive systems [58]. Skeletal muscle secretes Amyrel, a stress-inducible α-amylase, which hydrolyzes polysaccharides in the blood into maltose [59]. This mechanism appears to be critical for preserving protein quality in the central nervous system during stress [59]. We also found six amylase gene copies in the pig genome, three of which exhibited significant upregulation in obese subjects (**Fig. 2E**). Conversely, this gene family exhibits relatively minor expression changes in obese humans and mice. (**Fig. 2E**). This suggests that pig amylase genes may play a critical role in maintaining metabolic homeostasis under conditions of obesity.

The *ABCC4* gene family was significantly expanded in the pig genome (ten copies) (**Fig. 2F**). The ABC transporter family is involved in the transmembrane transport of various molecules. *ABCC4* expression increases with hepatic cholestasis and accumulation of liver toxins, suggesting on involvement in the efflux of toxic substances to protect the liver from damage [60]. Inhibition of *ABCC4* in cardiomyocytes leads to impaired cyclic adenosine monophosphate signaling (cAMP signaling, plays an important role in regulating energy metabolism [61]), which enhances myocardial contractility. However, prolonged inhibition of expression can cause cardiac hypertrophy [62]. Additionally, *ABCC4*-knockout mice exhibit typical obesity phenotypes, including adipocyte hypertrophy and insulin resistance, through the disruption of cAMP transport [63,64]. Although the function of the ABCC4 family is rarely reported in skeletal muscle, we found that three of the ten copies of the pig genome were significantly up-regulated in obese subjects (**Fig. 2F**). This suggests that the dynamic expression of these copies might optimize energy metabolism efficiency in pig skeletal muscle under obesity conditions by regulating the cAMP signaling and preventing the accumulation of lipotoxic substances through the transport of intra- and extra-cellular metabolites.

In summary, these results suggest that pigs may maintain myocyte or systemic metabolic homeostasis in an obese state through gene dosage effects or sub-functional differentiation.

### Obesity-induced divergent expression changes of single-copy genes across species

**Fig 3:**
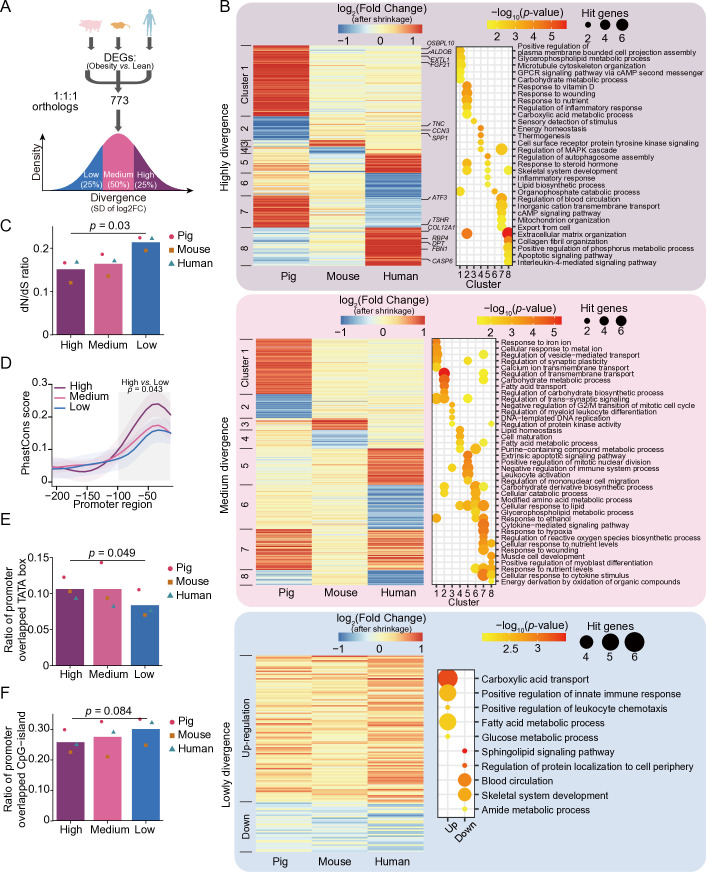
Cross-species comparison of expression changes of single-copy genes induced by obesity. **A**: Schematic of the identification of divergently expressed genes. **B:** Expression change profiles of genes with different divergence levels (left) and their functional enrichment results (right). **C:** Non-synonymous to synonymous substitutions (dN/dS) ratio of genes with different divergence levels. **D:** Conservation scores (PhastCons, human genome) of the 200-bp upstream promoter region of genes with different divergence levels. **E**–**F:** Proportion of promoter regions overlapping with the TATA box (**E**) and CpG islands (**F**) for each divergent gene. In panels **C**, **E** and **F**, different shapes represent the median values for each species, and the bar plots indicate the average values across species. The *p*-values in **C**, **E** and **F** were calculated by Student’s *t*-test, while the *p*-values in **D** were calculated by Wilcoxon rank sum test.

In addition to changes in gene copy numbers, the divergence of expression changes between species is also worth exploring. We identified obesity-induced DEGs in the fast-twitch muscle groups of pigs, mice, and humans, and selected 773 single-copy orthologous genes. Then, the DEGs were classified into high-, medium- and low-divergence expression changes, based on the standard deviation of the expression changes as a quantification of the divergent effects of obesity on skeletal muscle across species (**Fig. 3A**, see the **Materials and methods** for details).

Genes with high- and medium-divergence mainly exhibited species-specific expression changes (Fig. 3B, top and medium panel; S3–S4 Table). Pig-specific DEGs were enriched in three main functional categories: 1) metabolism-related pathways (Cluster 1 of the high-divergence genes), including ‘glycerophospholipid metabolism’, ‘cAMP signaling’, and ‘carbohydrate metabolism’, with genes in this cluster being specifically upregulated in obese pigs; 2) stress response pathways (Cluster 2 of the high-divergence genes), involved in ‘nutrient sensing’, ‘response to vitamin D’, and ‘inflammatory response’, with their expression levels specifically downregulated in obese pigs; and 3) transport-related pathways (Clusters 1 and 2 of the medium-divergence genes), including ‘fatty acid transport’, ‘ion transmembrane transport’, and ‘vesicle-mediated transport’ (Fig. 3B, top and medium panel; S3 Table). For example, *ALDOB*, *EXTL1*, and *OSBPL10* (involved in metabolism), as well as *FGF21* (a myokine, which protects from diet-induced obesity and insulin resistance [65,66]) were upregulated in the skeletal muscle of obese pigs (Fig. 3B; S4 Table, the biological function annotation of high divergent genes), whereas these genes have been reported to be downregulated in obese humans or mice [67–71]. Furthermore, *CCN3*, *SPP1*, and *TNC* (inflammatory- and immune-related genes) were downregulated in the skeletal muscle of pigs (Fig. 3B; S4 Table). However, previous studies have reported that these genes are upregulated in obesity at both the transcriptional and protein levels (S4 Table). In contrast, although genes in the Clusters 3–6 of high- and medium-divergent groups were species-specific DEGs, they participated in similar biological processes (Fig. 3B, top and medium panel; S3 Table). Human or mouse-specific upregulated DEGs (Clusters 3 and 5 of high- and medium-divergence groups) showed enrichment in immune and inflammatory responses (*e.g.,* ‘inflammatory response’, ‘leukocyte differentiation’, ‘leukocyte activation’), while human or mouse-specific downregulated DEGs (Clusters 4 and 6 of high- and medium-divergent groups) were mainly associated with metabolic processes (*e.g.,* ‘MAPK signaling’, ‘cellular catabolic process’, ‘fatty acid metabolic process’).

Notably, Clusters 7 and 8 of high-divergence genes showed an inverse pattern of change (Fig. 3B, top panel; S4 Table). Cluster 7, involved in energy metabolism-related pathways (*e.g.,* ‘cAMP signaling’, ‘mitochondrion organization’), was upregulated in obese pigs but downregulated in obese humans. Conversely, Cluster 8, enriched in stress response (*e.g.,* ‘apoptosis’, ‘interleukin signaling’ and ‘extracellular matrix organization’), was downregulated in obese pigs but upregulated in obese humans. For example, thermogenesis-related genes *TSHR* and *ATF3* were upregulated in obese pigs but downregulated in obese humans (Fig. 3B, top panel; S4 Table). In contrast, the adipogenesis genes *COL12A1*, *FBN1* and *RBP4*, as well as the genes *CASP6* and *DPT* that involved in inflammation or immune response, were downregulated in obese pigs but upregulated in obese human (Fig. 3B, top panel; S4 Table), consistent with previous reports showing their upregulation in obesity (S4 Table). Additionally, Clusters 7 and 8 of medium-divergence genes showed a conserved pattern of change in the pigs and humans (Fig. 3B, medium panel; S3 Table), and particularly in stress response pathways (*e.g.,* ‘hypoxia’, ‘cytokine signaling’ and ‘response to nutrients’). As expected, low-divergence genes showed a conserved expression changes across species (Fig. 3B, bottom panel; S3 Table), which mainly involved in pathways related to metabolism (*e.g.,* ‘lipid metabolism’ and ‘amino acid metabolism’) and stress response (*e.g.,* ‘immune response’ and ‘chemotaxis’).

These results suggest that the skeletal muscle of obese pigs exhibit enhanced expression of genes related energy metabolism and reduced inflammation and immune responses. This combination of a high metabolic capability and a low inflammatory burden may synergistically explain how pigs maintain skeletal muscle metabolic homeostasis under a high-fat diet.

To further investigate the evolutionary mechanisms underlying these obesity-induced divergence in expression changes, we examined the selective pressure and promoter features of the divergent genes. We found that most the divergent genes were under purifying selection (dN/dS < 1, a ratio of nonsynonymous to synonymous substitutions), indicating that these genes were functionally more important. Furthermore, the high-divergence genes exhibited stronger purifying selection (*p*-value = 0.03, Student’s *t*-test) (**Fig. 3C**), and higher conservation of their upstream promoter sequences (within 100 bp) compared to low-divergence genes (*p*-value = 0.043, Wilcoxon rank sum test) (**Fig. 3D**). Promoters that contain a TATA box tend to evolve at a slower rate, while CpG islands tend to be associated with rapidly evolving promoter sequences [72]. As expected, the promoters of high-divergence genes contained more TATA boxes and fewer CpG islands than those of low-divergence genes (Figs 3E–F). These findings demonstrate that the high-divergence genes were under stronger selective constraints, with their promoters enriched in TATA boxes and depleted in CpG islands.

### Regulatory landscape of the promoter region of obesity-induced divergent genes

Previous studies have shown that TATA-containing promoters tend to have a higher density of regulatory motifs [73]. To further explore the expression regulation of divergent genes, we downloaded ATAC-seq data from the fast-twitch muscles of healthy pigs, mice, and humans (S1 Table). After normalizing for length, we focused on the open chromatin regions (OCRs) within the promoters of divergent genes. These OCRs exhibit a greater degree of overlap with *cis*-eQTLs associated with the same gene (*p*-value < 0.05, permutation test), based on the eQTL data from the GTEx project for both humans and pigs (S3 Fig). This supports a potential regulatory relationship between these OCRs and divergent genes.

**Fig 4:**
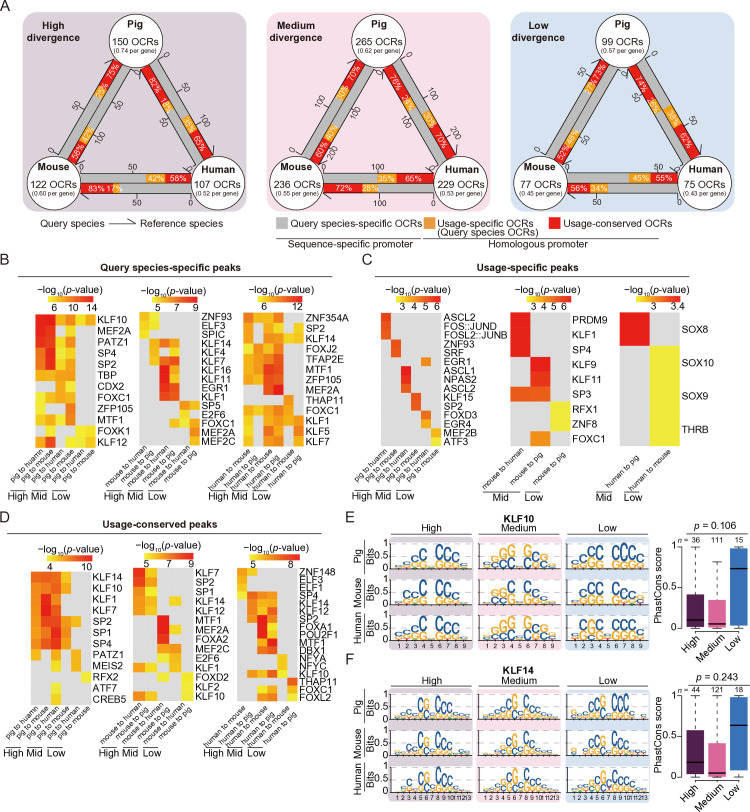
Chromatin accessibility differences of promoter regions of obesity-induced divergent genes. **A**: Classification of divergence-associated open chromatin regions (OCRs) based on synteny across species. **B**–**D**: Transcription factor enrichment of species-specific OCRs **(B)**, usage-specific OCRs (**C**) and usage-conserved OCRs **(D)**. **E**–**F**: The motif logo (left) and human PhastCons score (right) of KLF10 (**E**) and KLF14 (**F**) binding sites. The number of OCR is marked on each box. The *p*-values in **E** and **F** were calculated by Student’s *t*-test.

We found that the promoters of high-divergence genes showed more occupied OCRs (~0.62 OCRs per promoter) than low-divergence genes (~0.48 OCRs per promoter) (**Fig. 4A**). By aligning each OCR to the syntenic regions in the other species, we classified the OCRs into three categories: 1) species-specific OCRs, which could not aligned with homologous promoter regions in the reference species; 2) usage-specific OCRs, which aligned to the homologous promoter regions in the reference species but lacked OCRs; and 3) usage-conserved OCRs, which aligned to the homologous promoter regions in the reference species and contained OCRs (**Fig. 4A**). Notably, among the aligned OCRs, the high-divergence genes (~70%) showed a higher proportion of usage-conserved OCRs compared to the low-divergence genes (~60%). These results suggest that highly divergent genes may have undergone stronger selective constraint, while exhibiting greater regulatory plasticity.

To further identify potential transcription factor binding sites associated with divergent genes, we performed motif enrichment analysis on the OCRs of genes with obvious expression changes (|log_2_FC| > 0.3) (Figs 4B–D). These transcription factors showed greater differential expression between obese and lean subjects compared to randomly selected genes (S4A Fig). Given the potential for cross-tissue regulation mechanisms [74], we also examined their expression across multiple tissues (S4B–D Fig). We found that the divergent gene associated OCRs were mainly enriched with binding sites for Krueppel-like factor (KLF) family transcription factors and Specificity protein (SP) family transcription factors, particularly for genes with high- and medium-divergence (Fig 4B–D). Moreover, the expression changes of KLF and SP family genes were more pronounced than those of randomly selected genes (Fig S4E). KLF-family transcription factors play a key role in systemic metabolic homeostasis. For example, KLF10 and KLF14 are crucial transcription factors responsive to the energy status, such as high-fat diet, sugar intake, and fasting [75–79], and regulate multiple pathways involved in glucose and lipid metabolism [80,81]. SP-family transcription factors share a highly conserved carboxyl-terminal DNA binding domain with KLFs and also play a key regulatory role in lipid and glucose homeostasis [82]. Furthermore, we noticed that the binding sites of the PATZ1 transcription factor were specifically enriched in pig-specific high-divergence OCRs (Fig 4B). PATZ1 is involved in maintaining cell stemness [83], and its knockout induces immune cell activation [84–86]. This suggests that the downregulation of immune response-related genes in the skeletal muscle of obese pigs (Fig 1F) may be transcriptionally regulated by PATZ1. In contrast, binding sites for the transcription factors ELF3 and SPIC (involved in inflammatory responses [87–90]) and TFAP2 (related to lipid biosynthesis [91]) were enriched in the high divergence-related OCRs specific to mice and humans (Fig 4B).

Notably, although the transcription factor binding sites are syntenic across species, the rearrangement of motifs may lead to significant differences in the transcriptional regulation mechanisms [92–94]. We further assessed the conservation of KLF10- and KLF14-motif binding sites, which were consistently enriched in the usage-conserved OCRs across the three species (**Fig 4D**). The results indicate that the KLF10 and KLF14 binding sites associated with the high-divergence genes were slightly less conserved than for the low-divergence genes, although this difference was not statistically significant (Fig 4E–F). This trend may reflect an adaptive evolutionary mechanism. Pan-genomic analyses in *Arabidopsis thaliana* have revealed that even conserved non-coding regions contain genetic variation. These regions are enriched for stress-responsive transcription factors and are frequently located near genes involved in environmental interactions [95]. Similarly, studies in yeast have shown that certain imprecise (weak) motif occur within highly conserved regions [96]. These findings suggest that turnover of redundant transcription factor binding sites may enables species to fine-tune gene expression in response to environmental changes, thereby promoting regulatory flexibility and adaptive potential.

Overall, these results suggest that the transcriptional divergence in the response to obesity stress across species may be influenced by the dynamic chromatin accessibility and the spatiotemporal binding specificity of transcription factors.

## Discussion

This study reveals that the impact of obesity on skeletal muscle gene expression in pigs diverges from the impact in humans and mice. Through gene family analysis, we have identified expanded gene families in pigs that are upregulated in obese individuals. These genes are primarily involved in nutrient sensing and metabolic homeostasis regulation. In contrast, gene families that are contracted in humans and mice, mainly associated with transcription and oxidative metabolism, are downregulated in obese individuals. Furthermore, we have identified a set of orthologous genes involved in metabolism and inflammatory responses, for which the expression changes are divergent across species. Of these, high-divergent genes experience stronger selective constraints. By combining chromatin accessibility analysis, we have further characterized the features of the dynamic regulatory elements of the divergent gene promoter regions, and revealed high-divergent genes exhibiting greater transcriptional regulatory plasticity.

The molecular mechanisms underlying the relationship between obesity and metabolic dysregulation remain incompletely understood, but chronic inflammation may play a key role. In adipocytes, as fat storage increases (*e.g.,* during obesity), immune cells infiltrate into adipose tissue, leading to the secretion of proinflammatory cytokines [97,98]. These cytokines can induce systemic low-grade chronic inflammation and are closely associated with dysregulated lipid metabolism [97,98]. Consistent with the pro-inflammatory phenotype observed in hypertrophic adipocytes, we also found that complement system-related genes (*C1QC* and *C2*), and immune response marker genes (*CASP6* and *DPT*) were activated in the skeletal muscle of obese human and mice. This suggests that obesity may disrupt skeletal muscle metabolic function by activating a chronic inflammatory response, and recruiting immune cells from the bloodstream. Interestingly, these pro-inflammatory and immune activation-related genes, alongside complement system genes were downregulated in the skeletal muscle of obese pigs. This phenomenon parallels the reduced levels of immune cell populations, cytokines, and complement components observed in the circulation of hibernating animals [99], which accumulate substantial fat reserves prior to hibernation without developing insulin resistance or diabetes [100]. This reduced immune system investment may create a more immune-tolerant environment conducive to lipid deposition in pigs or hibernating animals, thus minimizing inflammatory responses. Moreover, it is noteworthy that genes related to lipolysis and thermogenesis were upregulated in the skeletal muscle of obese pigs, which may help obese pigs to metabolize excess fatty acids.

Various mechanisms contribute to phenotypic differences between species, including the evolution of non-orthologous genes (*e.g.,* rapidly evolving gene families) and orthologous genes (*e.g.,* changes in amino acid sequences that affect function). However, the evolution of gene regulation and its role in controlling gene expression remains less well understood. Our results show that genes that are highly divergent in response to obesity stress exhibit species-specific expression patterns, but these genes exhibit greater sequence conservation in both their coding and promoter regions. This paradox of sequence conservation and expression divergence is consistent across eukaryotic species [101–104]. Collectively, these studies highlight that genes with high transcriptional plasticity under stress tend to contain TATA box elements in their promoter regions. The TATA box is a conserved element in eukaryotes, playing a critical role in the assembly of the transcriptional machinery. Studies suggest that genes containing TATA box element show more dispersed transcription factor binding site distributions and exhibit variable expression under different stress conditions, while genes without TATA box typically display sharply defined transcription factor binding sites and are constitutively expressed [105,106]. This may be related to the characteristics of their promoter regions. TATA box genes tend to have higher proximal nucleosome occupancy, which increases their sensitivity to environmental changes [73,107]. Additionally, the transcriptional complex in these genes is often assembled at the promoter, enabling continuous initiation of transcription and again enhancing their responsiveness to environmental changes [108]. This may explain why, despite their higher nucleotide-level conservation, highly divergent genes exhibit greater transcriptional plasticity.

While our cross-species analysis provides valuable insights into divergent transcriptional responses of skeletal muscle to obesity cross species, certain limitations warrant consideration. First, in pigs and mice, obesity was induced by 22 and 10 weeks of high-fat diet, respectively, modeling relatively short-term metabolic stress. In contrast, human obesity typically develops over years, involving complex and chronic physiological adaptations. Although we prioritized divergence of expression change analysis to mitigate baseline disparities (e.g., lifespan, feeding habits and basal metabolic rates), the discrepancy in timescale may influence the gene regulatory landscape, potentially shifting the response from acute inflammation to chronic low-grade inflammation [109,110] and progressive tissue fibrosis [111]. Second, although we identified species-specific transcriptional changes in response to obesity, the precise biological functions of these genes remain to be experimentally validated. Moreover, our findings indicate potential regulatory divergence among species, however, the causal relationships between species-specific regulatory elements and their target genes remain unconfirmed. Elucidating these mechanisms will require future functional investigations, such as CRISPR-based perturbation assays. To facilitate future studies, we provide detailed annotations of the divergent genes (S4 Table), including literature-supported evidence for the involvement of highly divergent genes in metabolic, inflammatory, and immune-related biological processes. Notably, given the physiological and metabolic similarities between pigs and humans [13,14], the identification of pig-specific obesity-response genes offers a valuable opportunity to uncover novel regulatory mechanisms and therapeutic targets that may not be apparent in traditional rodent models. These findings may also help bridge the gap between experimental discovery and clinical translation in obesity research. Understanding the molecular basis of the pig’s adaptation to obesity-prone environments may ultimately yield insights into the development of therapeutic strategies for obesity-associated muscle dysfunction in humans.

In summary, we have identified a set of genes exhibiting divergent responses to obesity stress across pigs, mice, and humans by comparing gene expression changes between obese and lean subjects. We have demonstrated that both the evolution of gene families and transcriptional regulation play a key role in shaping these expression divergences. Additionally, we have characterized chromatin accessibility features in the promoter regions of the divergent genes. These rapidly evolving gene families, divergent genes, along with potential transcription factor binding sites, provide a foundational resource to guide future functional investigations into obesity-related gene regulation across species.

## Supporting information

S1 Table Summary of RNA-seq and ATAC-seq alignment information. (XLSX)

S2 Table Core gene annotation of obesity- associated gene sets. (XLSX)

S3 Table Functional enrichment analysis of genes with divergent responses to obesity. (XLSX)

S4 Table Cross-species transcriptional response landscape of divergent genes to obesity. This table summarizes the expression changes of divergent genes in response to obesity across human, mouse, and pig skeletal muscle. Genes exhibiting highly divergent responses among species were annotated with known biological functions and literature-based expression changes related to obesity.
(XLSX)

S1 Fig Influence of lowly expressed genes on interspecies log_2_ fold-change divergence and the effect of shrinkage. **A:** Distribution of log_2_FC for lowly and highly expressed genes before and after shrinkage. **B:** Interspecies divergence of log_2_FC for lowly expressed genes, with and without shrinkage. **C:** An illustrative example showing how a lowly expressed gene can contribute disproportionately to interspecies log_2_FC variation. **D:** Shrinkage of log_2_FC values using an empirical Bayes approach reduces variability introduced by low-expression genes. **E:** Pairwise correlations of interspecies log_2_FC divergence values calculated using different normalization methods, demonstrating the robustness of divergence estimates.
(TIF)

S2 Fig Transcriptomic profiles of obese humans and mice based on publicly available datasets. **A–C**: t-SNE analysis (**A**), volcano plot of differentially expressed genes (**B**), and functional enrichment analysis (**C**) based on transcriptomic data from a mouse model of obesity. **D–F**: t-SNE analysis (**D**), volcano plot of differentially expressed genes (**E**), and functional enrichment analysis (**F**) based on transcriptomic data from obese patients with T2D and healthy controls.
(TIF)

S3 Fig Enrichment of eQTLs in promoter-associated open chromatin regions (OCRs) of divergent genes. **A–B:** Enrichment of pig GTEx tissue-specific eQTLs in pig OCRs, with muscle-related eQTLs showing the highest enrichment (**A**); Pig muscle-related eQTLs more frequently target the same gene associated with the pig promoter-OCRs of divergent genes, compared to randomly selected 501 bp promoter regions (+2200 bp to −500 bp of TSS) (**B**). **C–D**: Enrichment of human GTEx tissue-specific eQTLs in human OCRs, with muscle-related eQTLs showing the second highest enrichment (**C**); Human muscle-related eQTLs more frequently target the same gene associated with the human promoter-OCRs of divergent genes, compared to randomly selected 501 bp promoter regions (+2200 bp to −500 bp of TSS) (**D**). **E:** Genomic distances between skeletal muscle eQTLs and their target genes in mice. Due to the inbred strain-cross design, the majority of mouse eQTLs identified were *trans*-acting, with target genes located on different chromosomes. Among *cis*-acting eQTLs (on the same chromosome), most were located more than 30 Mb from their target genes. eQTLs were identified using the R package qtl2 based on publicly available data from van Nas et al. (2010) (PMID: 20439777). These results highlight the need for future experimental validation to establish direct regulatory relationships between candidate OCRs and their associated genes.
(TIF)

S4 Fig Expression profiles of transcription factors significantly enriched in OCRs of divergent genes. **A:** Transcription factors enriched in OCRs of divergent genes in pigs, mice, and humans show greater expression changes between lean and obese subjects compared to 1,000 randomly selected genes. **B**: Tissue-specific expression of transcription factors enriched in OCRs of divergent genes in the pig, based on the pig GTEx dataset. **C:** Expression patterns of transcription factors in OCRs of divergent genes in the mouse, based on the EMBL-EBI Mouse Expression Atlas. **D:** Tissue-specific expression of transcription factors enriched in OCRs of divergent genes in humans, based on the human GTEx dataset. **E**: The KLF and SP transcription factors, whose binding motifs were most enriched in the promoters of divergent genes, exhibited differential expression between obese and lean subjects.
(TIF)
