# Supplementary figures and images for: Obesity induced transcriptional changes in skeletal muscle across different species
Source: PLoS One. 2025 Jul 14;20(7):e0327988. doi: 10.1371/journal.pone.0327988 (PMC12258593; doi:10.1371/journal.pone.0327988)

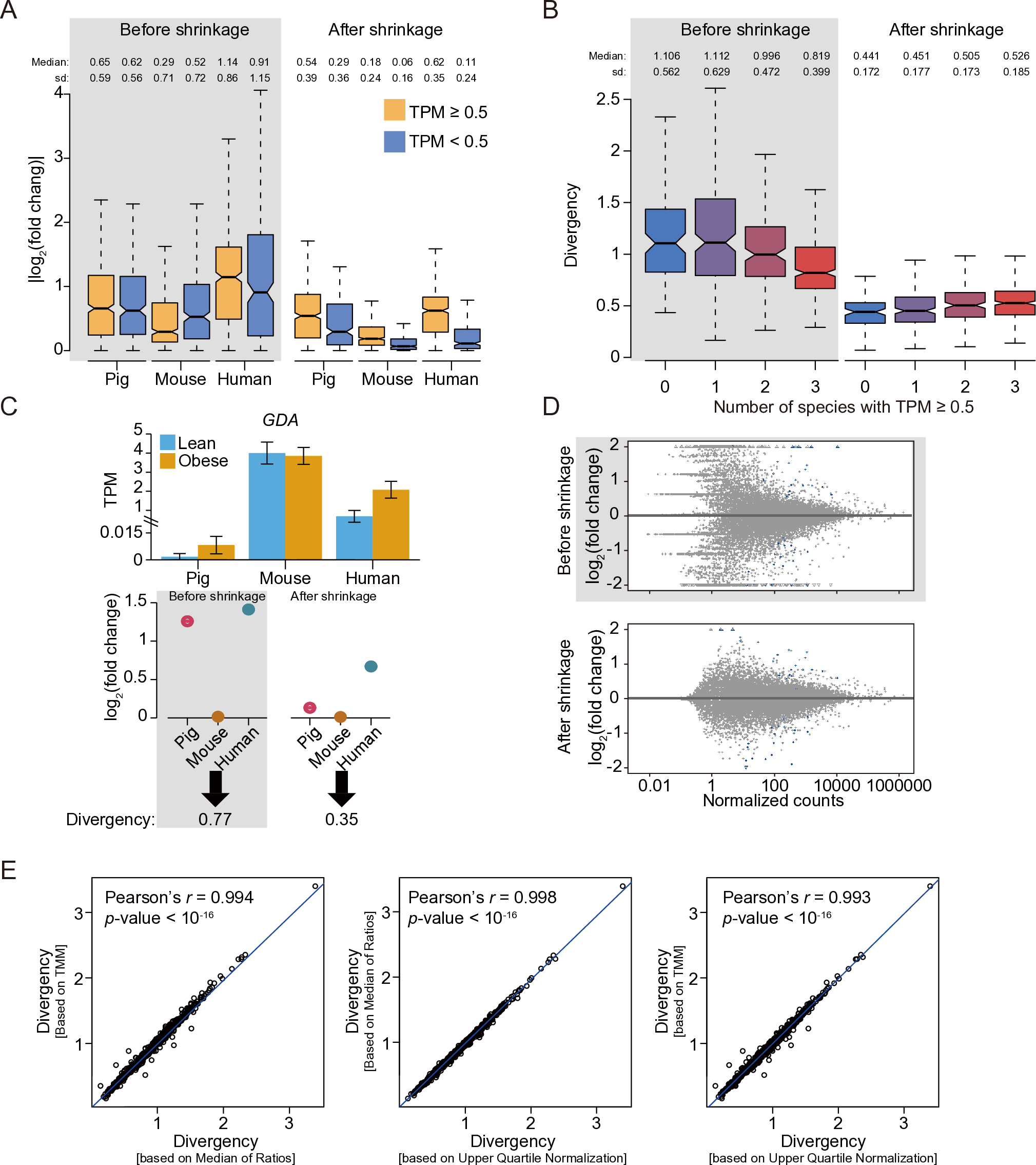

Supplement: S1 Fig — A: Distribution of log2FC for lowly and highly expressed genes before and after shrinkage. B: Interspecies divergence of log2FC for lowly expressed genes, with and without shrinkage. C: An illustrative example showing how a lowly expressed gene can contribute disproportionately to interspecies log2FC variation. D: Shrinkage of log2FC values using an empirical Bayes approach reduces variability introduced by low-expression genes. E: Pairwise correlations of interspecies log2FC divergence values calculated using different normalization methods, demonstrating the robustness of divergence estimates. (TIF) [file pone.0327988.s005.tif]

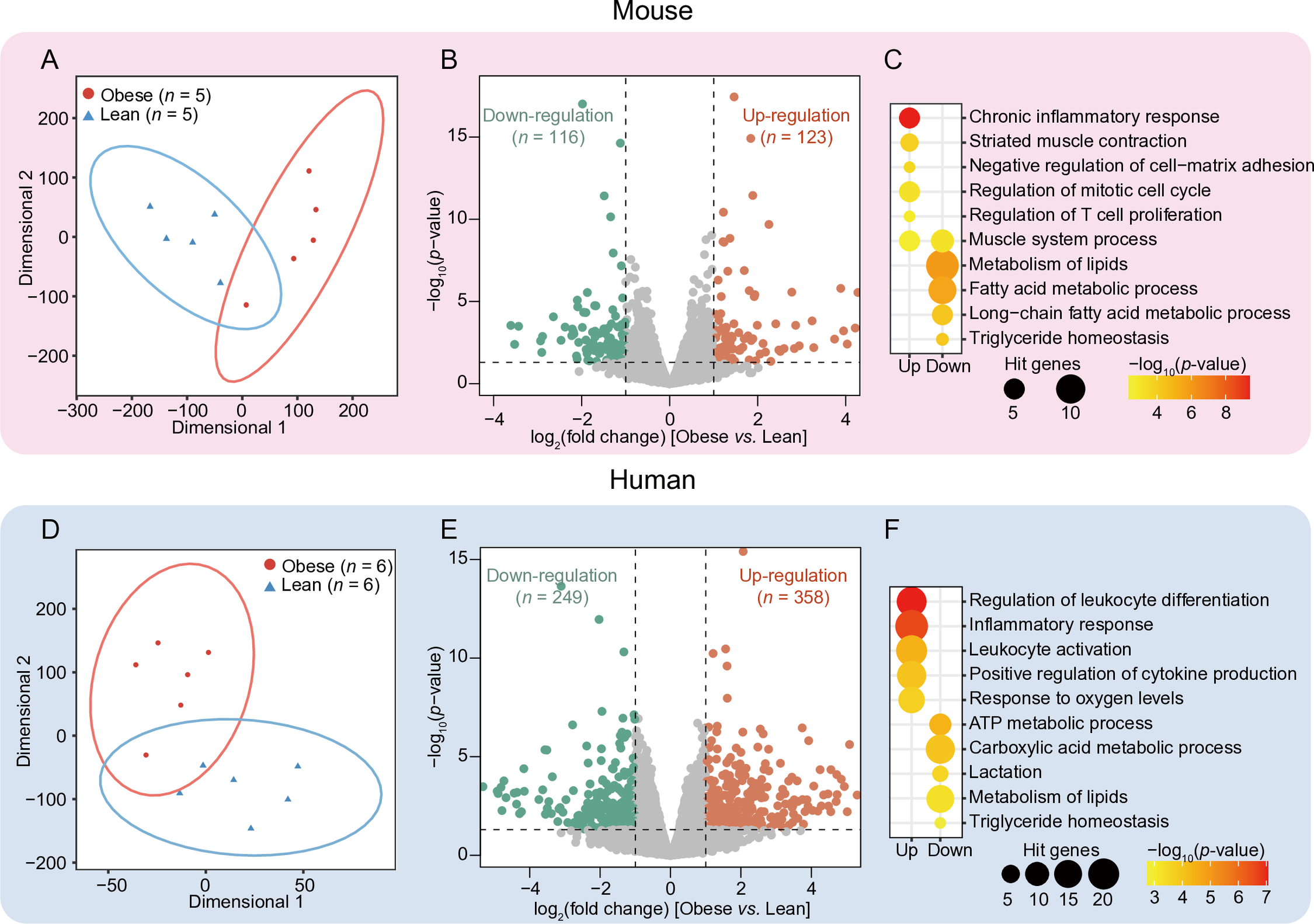

Supplement: S2 Fig — A–C: t-SNE analysis (A), volcano plot of differentially expressed genes (B), and functional enrichment analysis (C) based on transcriptomic data from a mouse model of obesity. D–F: t-SNE analysis (D), volcano plot of differentially expressed genes (E), and functional enrichment analysis (F) based on transcriptomic data from obese patients with T2D and healthy controls. (TIF) [file pone.0327988.s006.tif]

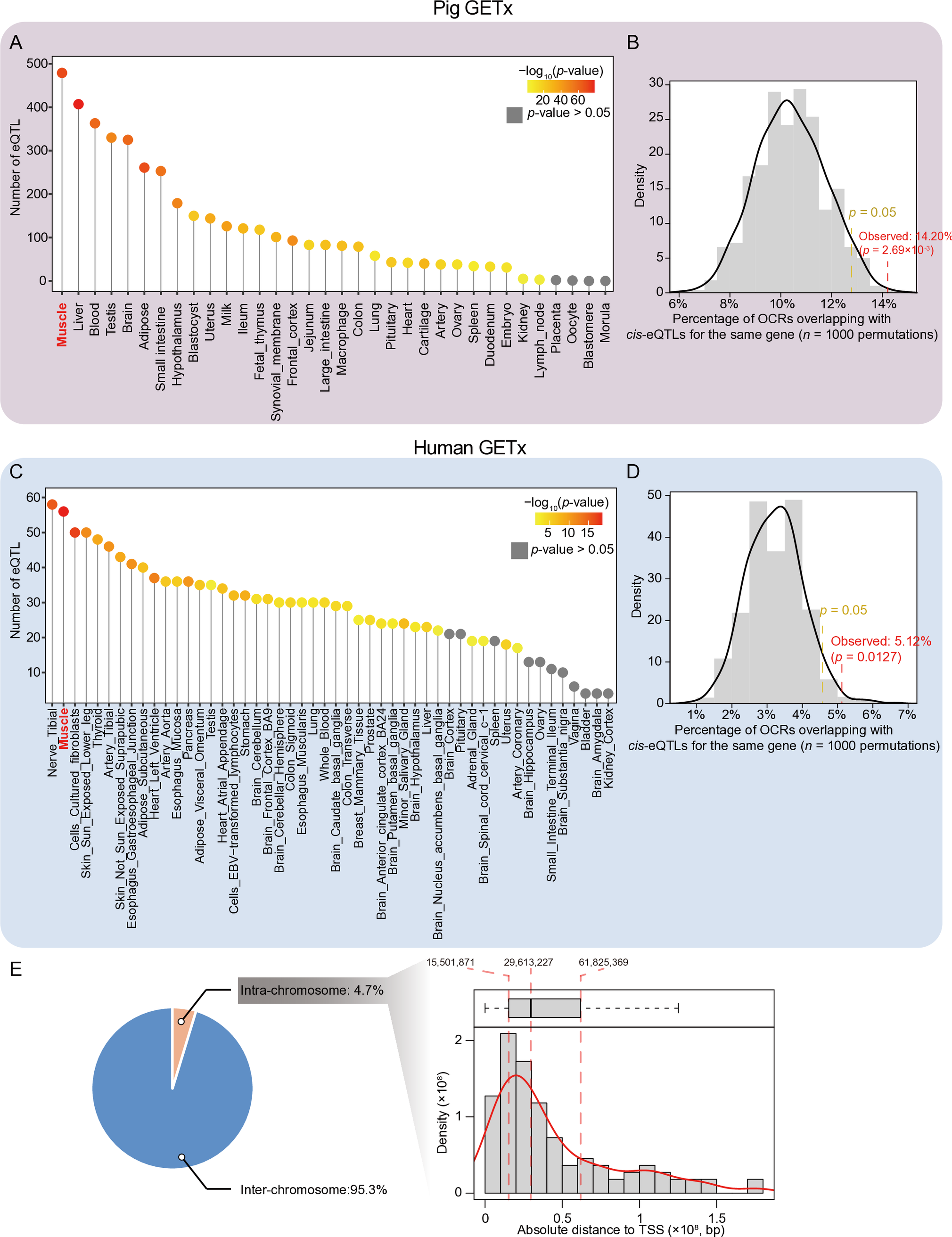

Supplement: S3 Fig — A–B: Enrichment of pig GTEx tissue-specific eQTLs in pig OCRs, with muscle-related eQTLs showing the highest enrichment (A); Pig muscle-related eQTLs more frequently target the same gene associated with the pig promoter-OCRs of divergent genes, compared to randomly selected 501 bp promoter regions (+2200 bp to −500 bp of TSS) (B). C–D: Enrichment of human GTEx tissue-specific eQTLs in human OCRs, with muscle-related eQTLs showing the second highest enrichment (C); Human muscle-related eQTLs more frequently target the same gene associated with the human promoter-OCRs of divergent genes, compared to randomly selected 501 bp promoter regions (+2200 bp to −500 bp of TSS) (D). E: Genomic distances between skeletal muscle eQTLs and their target genes in mice. Due to the inbred strain-cross design, the majority of mouse eQTLs identified were trans-acting, with target genes located on different chromosomes. Among cis-acting eQTLs (on the same chromosome), most were located more than 30 Mb from their target genes. eQTLs were identified using the R package qtl2 based on publicly available data from van Nas et al. (2010) (PMID: 20439777). These results highlight the need for future experimental validation to establish direct regulatory relationships between candidate OCRs and their associated genes. (TIF) [file pone.0327988.s007.tif]

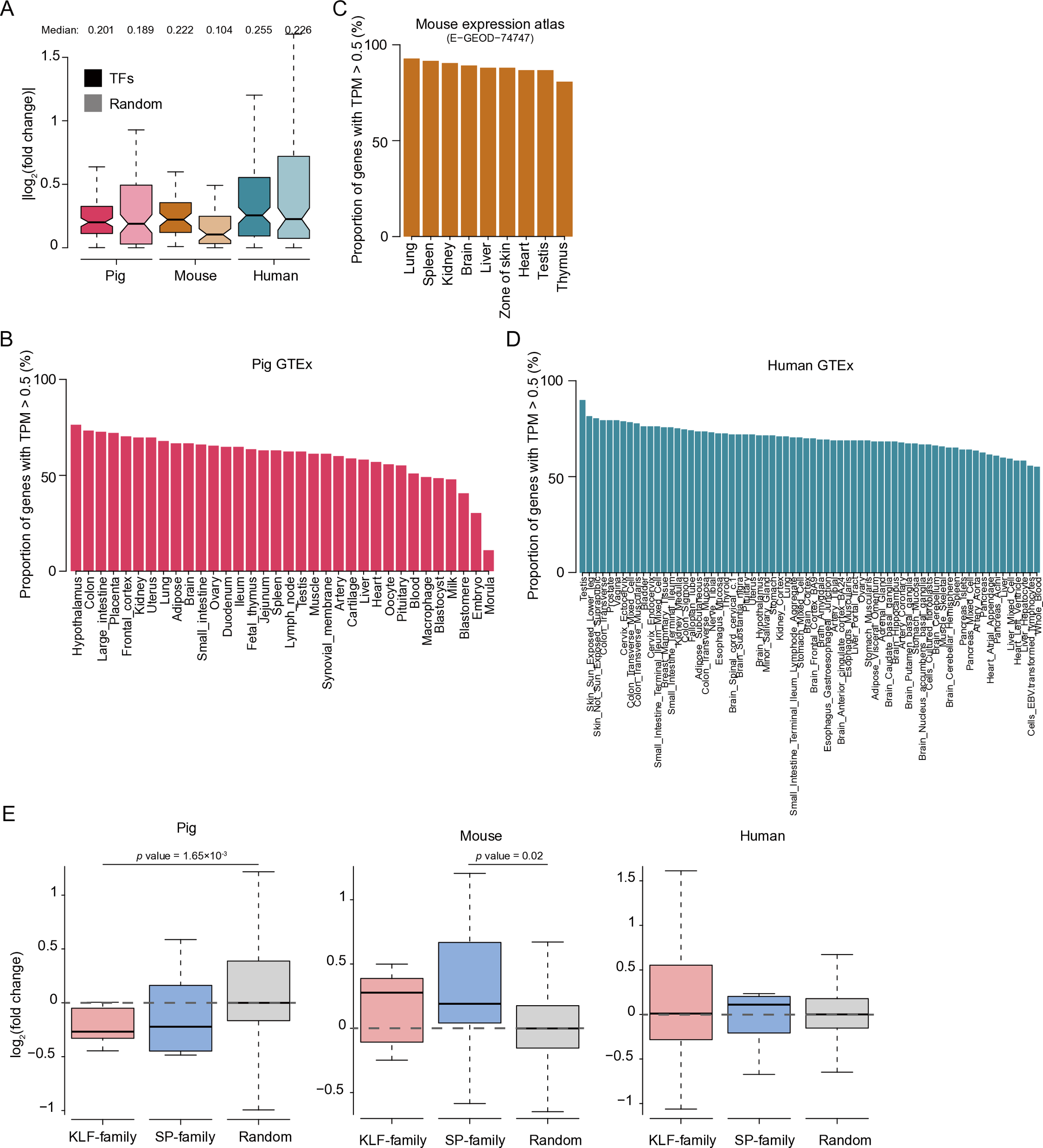

Supplement: S4 Fig — A: Transcription factors enriched in OCRs of divergent genes in pigs, mice, and humans show greater expression changes between lean and obese subjects compared to 1,000 randomly selected genes. B: Tissue-specific expression of transcription factors enriched in OCRs of divergent genes in the pig, based on the pig GTEx dataset. C: Expression patterns of transcription factors in OCRs of divergent genes in the mouse, based on the EMBL-EBI Mouse Expression Atlas. D: Tissue-specific expression of transcription factors enriched in OCRs of divergent genes in humans, based on the human GTEx dataset. E: The KLF and SP transcription factors, whose binding motifs were most enriched in the promoters of divergent genes, exhibited differential expression between obese and lean subjects. (TIF) [file pone.0327988.s008.tif]
